# Anti-Müllerian Hormone and Ovarian Reserve: Update on Assessing Ovarian Function

**DOI:** 10.1210/clinem/dgaa513

**Published:** 2020-08-08

**Authors:** Loes M E Moolhuijsen, Jenny A Visser

**Affiliations:** Department of Internal Medicine, Erasmus MC, University Medical Center Rotterdam, Rotterdam, The Netherlands

**Keywords:** ovarian reserve, AMH, functional ovarian reserve, menopause, fertility prediction, AMH assays

## Abstract

**Context:**

Anti-müllerian hormone (AMH) is produced by granulosa cells of small, growing follicles in the ovary. Serum AMH levels strongly correlate with the number of growing follicles, and therefore AMH has received increasing attention as a marker for ovarian reserve. This review summarizes recent findings and limitations in the application of serum AMH in ovarian reserve assessment.

**Evidence Acquisition:**

A PubMed search was conducted to find recent literature on the measurements and use of serum AMH as a marker for ovarian reserve.

**Evidence Synthesis:**

Serum AMH levels are measured to assess the “functional ovarian reserve,” a term that is preferred over “ovarian reserve,” since AMH levels reflect the pool of growing follicles that potentially can ovulate. Serum AMH levels are used in individualized follicle-stimulating hormone dosing protocols and may predict the risk of poor response or ovarian hyperstimulation syndrome but has limited value in predicting ongoing pregnancy. Serum AMH levels are studied to predict natural or disease-related age of menopause. Studies show that the age-dependent decline rates of AMH vary among women. The generalized implementation of serum AMH measurement has also led to an increase in diagnostic assays, including automated assays. However, direct comparison of results remains problematic.

**Conclusion:**

Serum AMH remains the preferred ovarian reserve marker. However, the lack of an international standard for AMH limits comparison between AMH assays. Furthermore, little is known about endogenous and exogenous factors that influence serum AMH levels, which limits proper interpretation of AMH values in a clinical setting.

## Introduction

**Figure 1.**
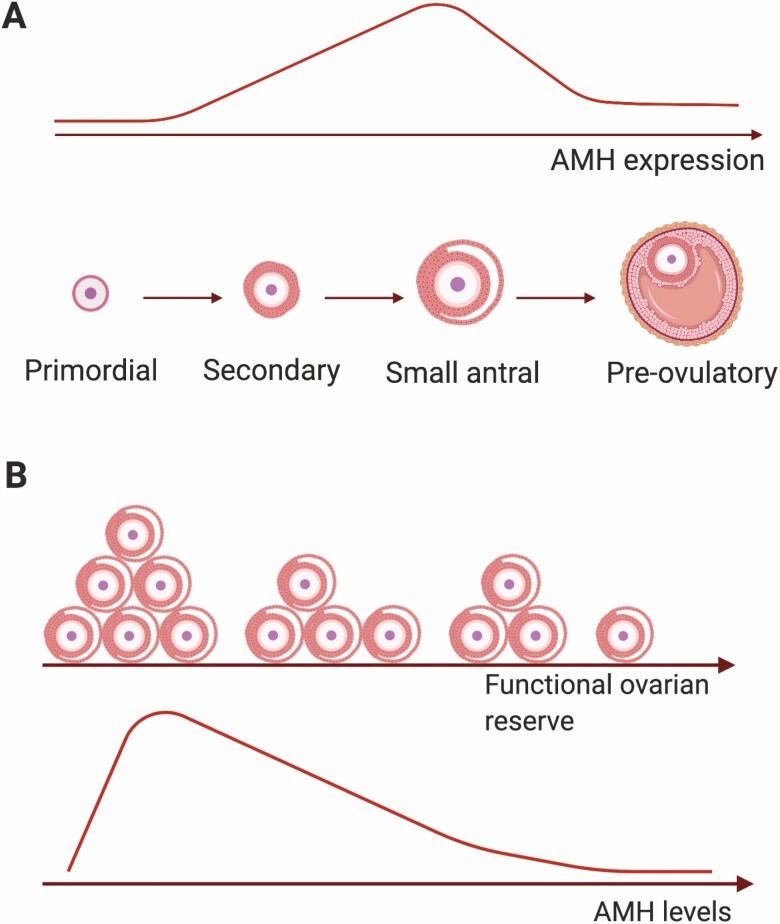
Anti-müllerian hormone expression and concentration in relation to folliculogenesis and ovarian reserve. (**A**) Anti-müllerian hormone (AMH) expression increases from the secondary stage onward until the small antral follicle stage. In preovulatory follicles, AMH is only expressed in cumulus granulosa cells surrounding the oocyte (dark pink layer). (**B**) With increasing age, the functional ovarian reserve decreases as a result of exhaustion of the primordial follicle pool. This leads to a decrease in the number of small antral follicles and consequently to a decrease in serum AMH levels, reaching undetectable levels at menopause. *Figure created with Biorender.*

Anti-müllerian hormone (AMH) is a member of the transforming growth factor beta family that has derived its name from its role during male sex differentiation by inducing the regression of the müllerian ducts. To date, AMH is best known as a serum marker for ovarian function, with assessment of AMH levels at both ends of the spectrum, that is, ovarian reserve and polycystic ovarian syndrome. In the ovary, AMH is expressed by granulosa cells of growing follicles from the primary up to the small antral stage. After follicle-stimulating hormone (FSH)-dependent selection, AMH expression disappears, although some expression remains in cumulus cells of preovulatory follicles. Also, in atretic follicles and corpora lutea, AMH expression is lost. This window of expression is largely preserved among species and in the adult human ovary. Increasing expression levels of AMH are detected in follicles up to 8 mm, and expression is absent in follicles >8 mm. This expression pattern is positively matched by AMH concentrations in follicular fluid, showing highest levels in follicles up to 8 mm and a sharp drop thereafter (Fig. 1A) (1).

Since AMH is expressed by growing follicles prior to FSH-dependent selection and has been shown to be detectable in circulation, serum AMH has taken momentum as a marker for ovarian function, in particular in the assessment of the quantitative aspect of the ovarian reserve, which is the focus of this review. By definition, the ovarian reserve is constituted by the quality and quantity of the primordial follicles, which both decline with increasing age (2). The number of growing follicles recruited from the primordial follicle pool reflect the number of primordial follicles. Since there is no serum marker that directly can measure the number of primordial follicles, a marker that reflects the number of growing follicles is currently the best proxy for the quantitative aspect of the ovarian reserve. Initial studies, performed nearly 2 decades ago, showed that serum AMH levels indeed strongly correlate with the number of growing follicles and that both decline with increasing age (3). Based on these initial studies, serum AMH was rapidly put forward as an indirect marker for the ovarian reserve despite limited knowledge of factors that regulate ovarian AMH expression and lack of standardized AMH assays.

Since serum AMH is only an indirect marker, this has led to confusion or even misinterpretation of the term ovarian reserve. To make a clear distinction between the pool of resting primordial follicles and the pool of growing follicles, the term functional ovarian reserve (FOR) has been suggested (4). FOR constitutes the pool of follicles 2 to 5 mm in diameter from which 1 follicle is destined to be selected by FSH and to ovulate (4, 5). This pool of growing follicles is known as the AMH-producing follicles, and thus serum AMH levels directly reflect FOR (Fig. 1B). In the clinical application of serum AMH to assess the ovarian reserve, it is therefore more accurate to use the term FOR. The importance to distinguish between ovarian reserve and FOR in the interpretation of AMH levels is illustrated by mouse studies and the scarce human studies in which the number of primordial follicles were determined. In mice, AMH levels remained constant at younger ages despite declining primordial follicle numbers. Only at older ages did AMH levels reflect the number of primordial follicles, while at all ages, serum AMH levels correlated with the number of growing follicles (6). Similar findings were observed in human studies in which the density of primordial and primary follicles was directly determined in ovaries removed because of benign gynecologic indications or prior to gonadotoxic therapies. In younger women AMH levels did not correlate, while in women of late reproductive age, a significant correlation was observed with the primordial follicle density (7-10). These studies suggest that at all ages serum AMH levels reflect FOR, and only at older reproductive ages, AMH levels may also reflect the ovarian reserve. Therefore, in this review, we will use the term FOR in order to discuss recent insights and limitations in the use of serum AMH to predict age of menopause in healthy women and in disease conditions.

## Serum AMH Levels in the General Population

Serum AMH levels are negatively correlated with age in adult women. However, studies aimed to develop normative data for AMH also showed that this correlation depends on the age category analyzed. From birth onward, AMH levels increased to plateau at approximately age 25 years (11, 12). Up to the age of approximately 16 years, AMH levels clearly were positively correlated with age. This positive correlation may reflect the increased rate of primordial follicle recruitment observed from birth up to approximately age 14 years (13). From age 25 years onward, AMH levels start to decline to undetectable levels at menopause, and only from this age onward, a negative correlation between AMH levels and age can be observed (11, 12). This pattern across ages appears consistent among different ethnicities (14-16). However, studies indicate that at any given age, there is a considerable variation in serum AMH levels (14, 17, 18). Thus, similar to what has been observed for antral follicle count (AFC), large interindividual variation exists for AMH levels (19, 20). Ethnicity may contribute to this variation and should be taken into account when interpreting AMH values. Although peak AMH levels at age 25 years were higher in Chinese women compared with European women, the age-related decline in Chinese women was greater leading to 28% and 80% lower AMH levels at age 30 and 45 years, respectively (21). In addition, African American women appeared to have lower serum AMH levels compared with White women but with a slower age-dependent decline (22, 23).

Serum AMH levels are generally measured during the early follicular phase, similar to other hormonal markers of ovarian function, such as FSH, estradiol, and inhibin B. However, it has been questioned whether the variations in serum AMH levels could be explained by differences during the menstrual cycle. While initial studies suggested that AMH levels are relatively stable during the menstrual cycle (24), more recent research suggest that AMH levels show significant intracycle variation up to 20.7% (25-27). Although the small number of individuals analyzed in these studies is a limitation, a clear pattern across the menstrual cycle, as evident for FSH or estradiol, was not present. Rather, the variation in AMH levels reflects the variation in AFC during the menstrual cycle according to a study by Overbeek et al in regularly cycling women (28). In this study, it was also shown that women with higher basal AMH levels, mostly younger women, had relatively higher variation in AMH levels across the menstrual cycle (28). Furthermore, studies observed that the intercycle variation in AMH can range from 28% to 163%, depending on the AMH assay used (27, 29). This intra-individual variation suggests that a single AMH measurement may lead to an inaccurate assessment of the FOR, which may have clinical consequences when the AMH value is used in an individualized ovarian stimulation protocol.

## Measurement of Serum AMH

**Figure 2.**
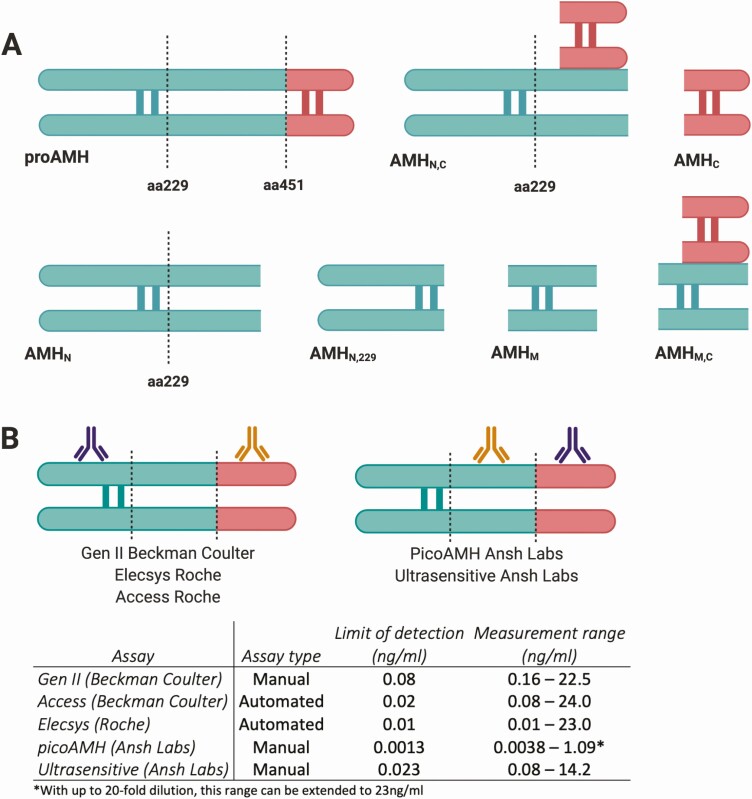
Potential anti-müllerian hormone (AMH) isoforms and AMH assay characteristics. (**A**) Illustration of the possible AMH isoforms present in the circulation. The black dotted lines represent the cleavage sites at amino acids 229 and 451, respectively. The N-terminal proregion is shown in green, and the C-terminal mature region is shown in red. (**B**) Depicts the characteristics of the different AMH assays. The capture antibody is shown in purple, and the detector antibody is shown in orange. The assay type, limit of detection, and the measurement range of the different AMH assays are shown in the table below. *Figure created with Biorender*.

AMH is produced as a 140-kDa disulfide-linked homodimer (proAMH), consisting of covalently bound monomers each of 560 amino acids (aa) (30). The AMH proprotein requires cleavages at its monobasic cleavage site at aa451 to generate an 110kDa N-terminal proregion (AMH_N_) dimer and a 25kDa C-terminal mature region (AMH_C_) dimer, which together form a stable noncovalently bound complex (AMH_N,C_) (31-33). In vitro studies using fetal rat testes and cell lines suggest that proteolytic cleavage of AMH occurs after secretion and is required to become biologically active (32). An additional cleavage site at aa229, of which the impact on AMH function remains to be determined, generates potentially 3 additional AMH isoforms, namely a shorter N-terminal peptide (AMH_N,229_), a mid-region (AMH_M_) and a mid-mature region (AMH_M,C_) (Fig. 2A).

The clinical importance of AMH measurement has led to the development of several AMH assays. Current frequently used manual assays include the modified Gen II assay (Beckman Coulter) and the ultra-sensitive AMH enzyme-linked immunosorbent assay (ELISA) and picoAMH assay (both Ansh Labs), which use different antibodies than the Gen II AMH assay (Fig. 2B). The picoAMH ELISA has an improved sensitivity in the lower range, resulting in a limit of detection of 1.3 pg/mL compared with 0.08 ng/mL for the Gen II assay (Fig. 2B). This difference in sensitivity may be of clinical importance when assessing serum AMH levels, particularly in cases where a low ovarian reserve is suspected. Furthermore, currently two automated AMH assays, Access AMH (Beckman Coulter) and Elecsys AMH (Roche), are available. These automated assays use the same antibody pair as the Gen II assay (Fig. 2B). Nevertheless, direct comparison of AMH values obtained by these assays is still problematic. Differences in values could be explained by the use of different antibody pairs (for the manual assays), which could lead to detection of different AMH isoforms. Sample instability or a matrix effect, which affects interlaboratory reproducibility, could contribute as well. The development of automated assays has increased assay precision, reproducibility, and speed of measurement, and therefore are superior over the manual assays.

Several studies have compared the manual and automated assays and present regression equations to allow comparison of serum AMH levels measured by different assays (34-38). However, as shown in Table 1, depending on the study and the level of AMH, the degree and the direction of conversion changes considerably. For instance, an AMH level of 1 ng/mL yielded a variation ranging from –12.8% to –25.2%, when comparing the Elecsys automated assay with the Gen II ELISA in the various studies. However, an AMH level of 5 ng/mL gives a variation ranging from –24% to +45% (34-36). Similar results were found when comparing the Gen II ELISA with the Access automated assay (36-38). The correlation varied from –9% to +34% and –19.4% to +7% for 1 ng/mL and 5 ng/mL AMH, respectively. Although the impact of this variation on absolute AMH values may not seem that large, these changes will impact AMH cutoff values and subsequently clinical decision-making in fertility treatment.

**Table 1.** Comparison of regression equations between anti-müllerian hormone assays

Paper	Regression Equation	Gen II ng/mL	Elecsys ng/mL (%)	Access ng/mL (%)
**Gassner et al, 2014**	Elecsys = 0.81*Gen II – 0.046	1	0.76 (–24%)	
		5	4.00 (–20%)	
**Hyldgaard et al, 2015**	Elecsys = 0.68*Gen II + 0.769	1	1.45 (+45%)	
		5	4.17 (–16.6%)	
**Nelson et al, 2015**	Elecsys = 0.73*Gen II + 0.087	1	0.82 (–18%)	
		5	3.74 (–25.2%)	
**Van Helden et al, 2015**	Elecsys = 0.88*Gen II – 0.039	1	0.84 (–16%)	
		5	4.36 (–12.8%)	
**Nelson et al, 2015**	Access = 0.78*Gen II + 0.128	1		0.91 (–9%)
		5		4.03 (–19.4%)
**Van Helden et al, 2015**	Access = 0.91*Gen II – 0.033	1		0.88 (–12%)
		5		4.52 (–9.6%)
**Pearson et al, 2016**	Access = 1.00*Gen II + 0.341	1		1.34 (+34%)
		5		5.35 (+7%)

AMH levels of 1 ng/mL and 5 ng/mL measured by the Gen II ELISA were converted using the regression equations of each study to provide values for the Elecsys and the Access automated AMH assays.

Abbreviations: AMH, anti-müllerian hormone; ELISA, enzyme-linked immunosorbent assay.

These differences between assays depending on AMH levels remain puzzling and contribute to conflicting results in clinical studies assessing AMH. The lack of an international AMH standard, even 20 years after the development of the first AMH ELISA assay, is one of the main problems hampering AMH assay comparison. Absence of uniformly calibrated assays limit the development of standardized AMH cutoff values needed to enhance patient safety and to prevent misinterpretation by clinicians unaware of this interassay variability.

The differences might be partly explained by a wide variation in population characteristics. Some studies have included women undergoing in vitro fertilization (IVF) treatment, whereas others included women undergoing laparoscopic sterilization or samples with an unclear clinical status related to fertility. Moreover, the sample size was relatively small (ranging from 23 women to 142 women), and also the age range was quite variable (23-56 years). Therefore, more research is needed to allow proper comparison of the different assays, using larger, clearly defined cohorts stratified by age.

In addition, in the development of an AMH international standard, it is important that the existence and clinical relevance of different AMH isoforms are unraveled. Current assays detect proAMH and AMH_N,C_. However, little is known about the presence and concentration of other AMH isoforms in the circulation. Although the AMH_C_ fragment is the biologically active form of AMH, Pankhurst et al suggest that it is not detectable in circulation (39). This might indicate that AMH_C_ is only generated after receptor binding of the AMH_N,C_ isoform, as shown in vitro (40). In a recent study by Wissing et al (41), it was shown that the amount of uncleaved AMH (proAMH) in normoovulatory women is only 3% of the total promature isoforms (proAMH and AMH_N,C_). This result was obtained using Ansh Labs ELISAs that either use a different antibody pair or sample treatment to distinguish the different isoforms (41). This suggests that the majority of serum AMH levels represents cleaved AMH. However, results should be interpreted with caution as matrix effects could alter the isoform conformation, limiting direct comparison of values.

Currently, it is unknown whether processing of AMH differs with age, by clinical condition, or even among women. Thus, it remains to be determined whether measurement of different AMH isoforms or their ratio has improved clinical relevance over total AMH, as assessed by current assays.

## Influencing Factors of Serum AMH

To properly interpret serum AMH levels, knowledge of factors that influence AMH levels is crucial. The majority of women of reproductive age use a type of hormonal contraceptive (HC), yet reported effects of HC use on serum AMH levels are conflicting. A systematic review by Amer et al (42), reassessing 15 studies, concluded that serum AMH levels in normoovulatory women decreased when HC was used for at least a year, and this effect was in the majority of the studies reversible after discontinuation of HC use. However, the extent of decline ranged from 14% to 55%, which could be explained by differences in type of HC use, duration of use, timing of AMH measurement during the menstrual cycle, and AMH assays used. Indeed, Landersoe et al (43) showed in a retrospective study that serum AMH levels were 30% to 40% lower in women using the oral contraceptive or the progesterone-only pill, while in women using an intrauterine device, only a decrease of 17% was observed. In addition, both studies reported a decline in AFC (42, 43), strongly suggesting that the change in serum AMH levels caused by HC use results from a change in follicle dynamics rather than a direct effect on AMH gene regulation. However, a direct effect of an altered gonadotropin and sex steroid milieu on AMH expression cannot be ruled out.

Several studies have identified body mass index (BMI) to negatively influence AMH levels. In a study by Moslehi et al (44) reanalyzing 26 studies, patients were subdivided into a fertile group without polycystic ovary syndrome (PCOS), an infertile non-PCOS group, and a PCOS group. The authors found a negative correlation between BMI and AMH in all groups, with a Fisher Z statistic of –0.15 (95% confidence interval [CI] –0.20 to –0.11) in the total population. However, BMI did not correlate with AFC, suggesting that BMI might directly affect AMH levels and not the FOR. Although the exact mechanism remains to be unraveled, leptin is thought to play a role. Merhi et al (45) demonstrated in cultured human granulosa cells, isolated from both small follicles (SFs; <14 mm) and large follicles (LFs; ≥14 mm), that treatment with recombinant leptin significantly suppressed AMH and AMH receptor II messenger ribonucleic acid (mRNA) levels. Treatment with a JAK2/STAT3 inhibitor prevented the leptin-induced downregulation of AMH mRNA expression, suggesting a direct involvement of the leptin signaling pathway. In contrast, a more recent study demonstrated that inhibition of leptin signaling through transfection of cultured human granulosa cells with leptin small interfering RNA (siLeptin) significantly reduced AMH secretion (46). It is plausible that leptin has different effects on AMH expression and secretion. Nevertheless, the precise mechanism remains to be elucidated.

Vitamin D (VitD) has increasingly been recognized to influence AMH levels. VitD levels exhibit seasonal variation with higher levels in summer compared with winter. Dennis et al (47) demonstrated that AMH levels in women of reproductive age also exhibit this seasonal variation, with levels being 18% lower in winter than in summer. In a subsequent study, healthy normoovulatory women were randomized to receive a single oral dose of 1,25-dihydroxy vitamin D (VD3), the active metabolite of VitD, or placebo (48). Within 24 hours after VD3 treatment, serum AMH levels sharply rose to 15.8 ± 1.1 nmol/L compared with 1.2 ± 0.7 nmol/L in control participants. However, the question remains whether VD3 increases serum AMH concentration directly via regulation of AMH expression or indirectly via a change in granulosa cell number. To address this question, Xu et al (49) investigated the effects of VD3 treatment on follicular development and AMH concentrations in macaques by culturing growing follicles in the presence of VD3. Analysis showed that VD3 increased preantral follicle survival, and AMH levels were significantly higher compared with controls (49). During the first 2 weeks of culture, VD3 treatment did not alter the follicular development or the hormonal milieu. However, during weeks 3 through 5, VD3 exposure increased antral follicle survival and AMH concentrations, while mRNA levels of AMH and AMH receptor II remained unchanged (50). These findings suggest that VD3 prevents granulosa cell apoptosis rather than directly regulating AMH expression, as also suggested by Merhi et al (51). In their study, VitD treatment did not affect AMH mRNA expression but rather inhibited AMH-induced signaling. This could lead to accelerated follicle maturation, which would explain the observed negative correlation between follicular fluid VitD levels and AMH mRNA expression (51). However, a direct effect on AMH expression cannot be ruled out since a VDR response element has been mapped to the AMH promoter (52).

These studies suggest that when counseling women on their FOR based on AMH levels, insight into factors that influence AMH expression but also follicle dynamics is important. It remains to be determined whether changes in AMH expression also lead to changes in number of primordial follicles, that is, the ovarian reserve, and whether such changes are in the same direction. This emphasizes the use of FOR over ovarian reserve in relation to AMH assessment.

## Use of Serum AMH Levels in the Prediction of Age of Menopause

In the Western world, the age at which a woman decides to have her first child has increased, and thereby also the risk of age-related involuntary infertility (53). Given the strong correlation between the age-related decline in primordial follicle number, number of growing follicles, and serum AMH levels, several studies have investigated whether serum AMH could aid in the prediction of age of menopause. A meta-analysis by Depmann et al (54), in which AMH levels from 6 studies were reanalyzed, concluded that serum AMH can predict time to menopause. However, compared with a woman’s age, the added value of serum AMH was limited as the C statistic only increased from 84% to 86%. Furthermore, serum AMH appeared to have limited precision on an individual level. Conflicting results have been reported in the prediction of onset of menopause in women of late reproductive age. In a recent population-based study, of which the majority of women were overweight or obese, it was shown that women aged 45 to 49 years with undetectable AMH levels had a 60% probability to become menopausal within 5 years (55). Furthermore, AMH did improve the prediction of menopausal onset compared with age alone (C statistic 91% vs 83%) (55). A recent multiethnic study, which included 1537 pre- or early perimenopausal women at baseline and with follow-up until 12 months of amenorrhea was reached (SWAN study), analyzed the prediction of the final menstrual period (FMP) by AMH levels. Although AMH was serially assessed in a small subset of this cohort, multiple samples of individual women were independently used in the statistical models to predict FMP. Combined with age and BMI, AMH had a better predictive value for FMP than FSH. In women younger than age 48 years, an AMH value <10 pg/mL had a 51% positive predictive value to predict reaching FMP within 12 months, which increased to 78% when reaching FMP within 36 months. In women aged ≥51 years, these values were 79% and 97%, respectively. Importantly, extending prediction of FMP within 12 months to within 36 months decreased the sensitivity of AMH significantly. In contrast, in women aged <45 years, an AMH <10 pg/mL had a low sensitivity and low positive predictive value in prediction FMP (56). Thus, combined these 2 latter studies suggest that in women of late reproductive age, assessment of AMH may aid in the prediction of age of menopause. However, it can be argued that prediction of age of menopause at a younger age is clinically more relevant for an individual woman, as is prediction of early menopause, that is, menopause before the age of 45 years.

In the above-discussed meta-analysis by Depmann et al (54), compared with age alone, AMH increased the C statistic from 52% to 80% in the prediction of early menopause. In a recent prospective study with a nested case-control design containing 327 cases, the use of serum AMH to predict early menopause was confirmed. A decrease of 0.10 ng/mL in AMH increased the risk of early menopause by 14% (95% CI, 1.10-1.18). Compared with an AMH level of 2.0 ng/mL, the odds ratio (OR) for early menopause was 23 for women with an AMH level of 0.5 ng/mL (57).

Most prediction models are based on a single AMH measurement and assume a comparable decline pattern in each woman. Recent studies analyzing longitudinal AMH measurements suggest that AMH may not follow a uniform decline trajectory. Analysis of the population-based Doetinchem cohort study with data available from 5 visits over a 20-year follow-up period, showed an age-dependent decline in AMH levels, which varied significantly for individual women. Furthermore, it was shown that the decline rate changed with age, accelerating after the age of 40 years (58). Hence, it has been suggested that using individual AMH decline patterns may improve the prediction of age of menopause. However, reanalyzing data of 2432 women from the Doetichem cohort study showed that an AMH decline rate alone, or in combination with age-specific AMH, had little additional value (59). In contrast, an Iranian study analyzing longitudinal data of 959 women during a follow-up of 14 years, of whom 55% reached menopause, did show that serial measurements of AMH improved the prediction of age of menopause, since addition of AMH decline rate to the model increased the C statistic to 78% compared with 70% for AMH alone (60). In a smaller subset of this cohort (n = 266) with shorter follow-up (average of 6.5 years with 3-year intervals), the authors previously confirmed that the decline rate of AMH was specific for each woman (61). Importantly, the authors also showed that the decline rate was dependent on age, which raises the question to at which age interval and how frequently AMH should be measured to accurately predict age of menopause.

Although the study of Ramezani Tehrani et al (60) did not specifically analyze prediction of early menopause, the authors did show that the predictive added value of an AMH decline rate was consistent when analyzing women younger or older than age 40 years. Based on their model, women with an AMH value of 0.1 ng/mL at the age 30 years have a predicted median age of menopause of 43.18 (37.56-46.33) years with a fifth percentile AMH decline rate, while with a 95th percentile decline rate, this is predicted at 33.63 (29.25-36.08) years (60). Similar to age of menopause, in the statistical models of de Kat et al (59), an AMH decline rate did not improve prediction of early menopause. In fact, in women younger than age 30 years, AMH levels may actually underestimate the risk of early menopause (59). While this outcome seems to contradict the studies discussed above, when validated, it may have clinical consequences since, particularly, women of this age category may deliberate whether or not to delay childbearing.

Based on current studies, the predictive value of serum AMH for age of menopause remains controversial. The majority of these studies have analyzed different age ranges, duration of follow-up, and AMH assays, making direct comparison of studies difficult. It also remains unclear whether current results obtained in regularly cycling women can be translated to infertile women in whom the ovarian reserve may be compromised. The potential impact of ethnicity on AMH decline rates also has not been analyzed in much detail. Thus, validation studies that incorporate additional variables are required to determine specific AMH thresholds in the prediction of age at menopause.

## Use of AMH Levels in the Prediction of Response to Controlled Ovarian Stimulation

Previous studies have also shown that AMH levels may aid in the prediction of ovarian response to controlled ovarian hyperstimulation (COH) protocols. Low AMH levels are correlated with a low response, defined as retrieval of less than 5 oocytes or cycle cancellation. Currently, 2 different ovarian stimulation approaches are widely used: a gonadotropin-releasing hormone agonist or a gonadotropin-releasing hormone antagonist in combination with recombinant or urinary FSH (62). To improve the response to COH, algorithms are used to calculate the individualized dosage of FSH. Recently, serum AMH measurement has been added to the list of factors, which include age, BMI, duration of subfertility, basal FSH, and AFC. The algorithms that include measurement of FSH, AFC, and AMH are called the ovarian reserve tests (ORTs).

Accurate and reliable calculation of the individual dosage is important since both under- and overstimulation could lead to cycle cancellation. In addition, an excessive response could result in the development of ovarian hyperstimulation syndrome, a potentially life-threatening condition. It is, however, unclear whether the use of these clinical characteristics significantly improves the prediction of ovarian response and clinical outcomes. Broer et al performed 2 meta-analyses to investigate the added value of ORTs to the patients’ characteristics of age, BMI, and duration of subfertility (63, 64). Of these 6 patient characteristics measured, AMH and AFC had the highest accuracy in predicting excessive ovarian response, defined as the yield of more than 15 oocytes, and in predicting poor ovarian response. The receiver-operation characteristic regression analysis for predicting an excessive response showed an area under the curve (AUC) of 0.81 (95% CI, 0.76-0.87) for AMH and 0.79 (95% CI, 0.74-0.84) for AFC, respectively. Combining these 2 tests slightly improved the model (AUC 0.85). In predicting a poor ovarian response, comparable results were found with an AUC of 0.78 (95% CI, 0.72-0.84) and 0.76 (95% CI, 0.70-0.82), respectively (64). Based on these studies, AMH and AFC are the best parameters to predict poor and excessive ovarian responses to COH.

The question remains whether individualizing treatment based on these parameters also improves clinical outcomes. Based on the studies from Broer et al, both AMH and AFC showed a very low predictive value for pregnancy rate after IVF, with an AUC of only 0.50 and 0.55, respectively (63, 64). A more recent meta-analysis further investigated this finding by reassessing 20 randomized controlled trials (65). In agreement with the studies from Broer et al (63, 64), the authors concluded that changing the dosage of stimulating medication based on individual ORTs, including AMH, does not significantly increase the chances on pregnancy and live birth.

Friss Petersen et al (66) investigated the effect of AMH alone in an individualized algorithm to dose FSH on the intended oocyte retrieval (5-14 oocytes) and clinical outcomes of patients undergoing IVF. Comparison of an AMH-based dosage of FSH with a standard dosage showed that the percentages of unintended responses (<5 oocytes or >15 oocytes) were comparable as were the clinical outcomes in terms of pregnancy rates and live birth. Hence, although AMH is a good predictor for ovarian response to COH, it does not improve the pregnancy rate and rate of live birth. However, an important note is that in women predicted to have an excessive response, individualized treatment based on ORTs did result in a slight decrease in the chance of developing ovarian hyperstimulation syndrome with an OR of 0.58 (95% CI, 0.34-1.00) based on 4 studies (65).

Drawing conclusions based on these findings remains complicated as the majority of these studies have used different cutoff values for ORTs and used different AMH assays. Therefore, the clinical application of AMH levels in ORT-based dose adaptation still needs to be demonstrated.

## Assessment of the FOR in Autoimmune Diseases

Women with autoimmune diseases are at risk for early menopause and therefore at increased risk for infertility and menopause-associated diseases such as osteoporosis and cardiovascular diseases. Hence, several studies have analyzed serum AMH levels to evaluate FOR in women with autoimmune diseases.

A recent meta-analysis of 19 studies analyzing patients with systemic lupus erythematosus concluded that serum AMH levels were significantly lower in patients with systemic lupus erythematosus compared with healthy control participants (pooled standardized mean differences −0.79; 95% CI, −1.41 to −0.18). In addition, AMH levels were further lowered by the immunosuppressant cyclophosphamide (pooled standardized mean differences of −0.58; 95% CI, −0.87 to −0.30) (67).

Women with recent-onset rheumatoid arthritis (RA) had comparable AMH levels with healthy control participants, and methotrexate use did not affect AMH levels (68). However, in a subsequent study, lower preconception AMH levels were observed in women with RA attempting to become pregnant. The authors suggested that disease duration might explain this difference. In agreement, other studies also reported 1.3-fold lower AMH levels in women with RA having a disease duration of >6 years (69, 70). In patients with juvenile idiopathic arthritis, nearly 2-fold lower AMH levels were measured (71).

In women with autoimmune thyroid disease (ATD), conflicting results have been reported. Adolescent girls newly diagnosed with ATD had either normal or higher AMH levels compared with age- and BMI-matched control participants (72-74). In adult women, the majority of studies reported modest but significantly lower AMH levels in women with thyroid dysfunction or confirmed ATD, although 1 study observed increased AMH levels (75). A direct relationship between altered thyroid hormone levels and AMH levels in ATD with or without supplementation with levothyroxine remains, however, uncertain due to conflicting results (76-81). If ATD affects FOR, the degree seems modest at best. However, an impact on COH outcome cannot be ruled out yet. Although the presence of ATD did not further worsen the response to COH in women with low AMH levels, it did impair COH response in women with normal AMH levels (82).

Women with type 1 diabetes (T1D) are suggested to have an earlier age at menopause (83), although this has been questioned more recently. In women with T1D older than age 33 years, 2-fold lower AMH levels were observed, and also the percentage of having AMH levels in the menopausal range was nearly 5-fold higher compared with control participants (84). In contrast, although lower AMH concentrations also were observed in a large community-based population cohort of women with T1D, this was only in women younger than age 35 years (85). Finally, in a cross-sectional, patient-control study, no differences in AMH levels, percentage of menopausal range of AMH levels, nor age of menopause were observed (86, 87). These inconsistent results could in part be explained by the fact that T1D also has a high prevalence of PCOS, which is associated with elevated AMH levels. The presence of women with combined PCOS and T1D therefore potentially may have masked effects on AMH levels.

Overall, these recent studies suggest that the relationship between autoimmune diseases and diminished FOR, as assessed by AMH, remains inconsistent. Additional well-controlled studies are needed to analyze the impact of disease onset, duration, and therapy on AMH levels.

## Assessment of the FOR After Cancer Treatment

Chemotherapy is known to have adverse effects on ovarian function and increases the risk of primary ovarian insufficiency (POI) posttreatment. In recent years, a number of studies have analyzed pre- and posttreatment AMH levels in patients with cancer, particularly in breast cancer, being the most common cancer in women worldwide. Studies agree that upon chemotherapy, AMH levels rapidly decline to (nearly) undetectable levels (88, 89). This rapid decline can be explained by the immediate elimination of AMH-producing growing follicles. Subsequent recovery of ovarian function varies among women. To improve counseling of patients with cancer for their future fertility, pre- and posttreatment AMH levels have been studied to identify women at risk for failure of ovarian recovery. Patients with breast cancer who developed POI had lower pretreatment AMH levels than those who resumed menses (89-91). Indeed, pretreatment AMH levels were predictive for chemotherapy-induced POI in patients who were premenopausal with breast cancer. In the study by Anderson et al (89), a cutoff pretreatment AMH level of <7.3 pmol/L (1.022 ng/mL) yielded an AUC of 0.77 with a sensitivity of 95% and specificity of 49%, while in the study of Xue et al, an AMH cutoff value of 0.965 ng/mL yielded a slightly higher AUC of 0.84 with a sensitivity of 74% and specificity of 82% (90, 91). Likewise, pretreatment AMH levels may predict ovarian recovery, expressed as an AMH level ≥1 ng/mL at 12 months postchemotherapy (adjusted OR 1.659; CI 95%, 1.261-2.182), although the detected value was modest after 2 years follow-up (adjusted OR 1.275; CI 95%, 1.141-1.426) (92). However, individual differences in ovarian recovery may limit the predictive value of AMH. Decantere et al showed that young patients with breast cancer who resumed menses by 6 months posttreatment showed an earlier and faster increase in AMH levels than those with a slow recovery, despite having similar pretreatment AMH levels and receiving the same chemotherapy protocol (93). Interestingly, in the slow recovery group, posttreatment AMH levels were lower compared with the fast recovery group (93). Indeed, lower posttreatment AMH levels also increased the risk of chemotherapy-induced POI (89, 94). Although the predictive value of AMH appears independent of age, studies agree that women younger than age 40 years have higher posttreatment AMH levels (89, 94). Cancer survivors seems to have a similar AMH decline rate as control women despite having lower posttreatment AMH levels (95). This suggests that in cancer survivors the exhaustion of FOR is not accelerated. However, predicting their reproductive life span remains challenging and will require additional longitudinal studies controlled for cancer type, age of cancer diagnosis, and treatment regimen. A recent study by Su et al (96), analyzing survivors of breast cancer, thyroid cancer, and lymphoma is one of the first studies starting to address these limitations. AMH trajectories indeed differed based on the gonadotoxic effect of the treatment. While treatment at younger age resulted in higher AMH trajectories, this protective effect was nullified upon treatment with high gonadotoxic agents (96).

In women treated for differentiated thyroid cancers, radioactive iodine (RAI) treatment has been suggested to induce early menopause. Although the impact of postsurgical RAI treatment on AMH levels seems less severe than chemotherapy, AMH levels did declined by at least 50% and only showed a partial recovery (97-99). Furthermore, the impact of RAI treatment on AMH levels was more pronounced in patients aged >35 years (97, 99). Thus, similar to patients with breast cancer, the ovarian reserve may be relatively protected when thyroid cancer is diagnosed at a younger age. However, given the strong decrease in AMH levels in younger women, also for thyroid cancer, additional prospective studies with sufficient follow-up are required.

## Conclusion

The improved sensitivity and automation of AMH assays has strengthened the role of serum AMH levels as a marker for the FOR. Assessment of serum AMH levels in the prediction of ovarian response to COH and age of menopause, being either natural or iatrogenic, may be useful. However, limitations have also surfaced. For proper interpretation of AMH levels, more knowledge is needed on endogenous and exogenous factors that regulate AMH expression. Studies further suggest that heterogeneity exists in AMH trajectories, which may hamper application in personalized patient counseling. Over the last years, the number of assays for AMH measurement has increased. However, differences exist between assays. An international standard for AMH is therefore urgently needed to establish assay-independent cutoff values.

## Data Availability

Data sharing is not applicable to this article as no datasets were generated or analyzed during the current study.

## Abbreviations

aa: amino acid
AFC: antral follicle count
AMH: anti-müllerian hormone
AMH_C_: 25kDa C-terminal mature region
AMH_N_: 110kDa N-terminal proregion
ATD: autoimmune thyroid disease
AUC: area under the curve
BMI: body mass index
CI: confidence interval
COH: controlled ovarian hyperstimulation
ELISA: enzyme-linked immunosorbent assay
FMP: final menstrual period
FOR: functional ovarian reserve
FSH: follicle-stimulating hormone
HC: hormonal contraceptive
IVF: in vitro fertilization
OR: odds ratio
ORT: ovarian reserve test
PCOS: polycystic ovary syndrome
POI: primary ovarian insufficiency
proAMH: 140-kDa disulfide-linked homodimer
RA: rheumatoid arthritis
RAI: radioactive iodine
RNA: ribonucleic acid
T1D: type 1 diabetes
VD3: 1,25-dihydroxy vitamin D
VitD: vitamin D

## Additional Information

***Disclosure Summary:*** L.M.E.M. has no conflict of interest. J.A.V. has received royalties from AMH assays, paid to the institute/lab with no personal financial gain.
